# Rationale, design, and baseline characteristics of the Acetylcystein for Contrast-Induced nephropaThy (ACT) Trial: a pragmatic randomized controlled trial to evaluate the efficacy of acetylcysteine for the prevention of contrast-induced nephropathy

**DOI:** 10.1186/1745-6215-10-38

**Published:** 2009-06-04

**Authors:** The ACT Trial Investigators

**Affiliations:** 1 Research Institute – Hospital do Coracao (HCor), Rua Abilio Soares 250, 12 floor, 04005-000 – São Paulo – SP, Brazil

## Abstract

**Background:**

Aceltylcysteine has been evaluated in several small trials as a means of reducing the risk of contrast-induced nephropathy (CIN), however systematic reviews of these studies do not provide conclusive answers. Therefore, a large randomized controlled trial (RCT) is needed to provide a reliable answer as to whether acetylcysteine is effective in decreasing the risk of CIN in high-risk patients undergoing angiographic procedures.

**Methods:**

ACT is a RCT of acetylcysteine versus placebo in 2,300 patients at-risk for CIN undergoing an intravascular angiographic procedure. The randomization list will be concealed. Participants, health care staff, investigators and outcome assessors will be blinded to whether patients receive acetylcysteine or placebo. All analysis will follow the intention-to-treat principle. The study drugs (acetylcysteine 1200 mg or placebo) will be administered orally twice daily for two doses before and two doses after the procedure. The primary outcome is the occurrence of CIN, defined as a 25% elevation of serum creatinine above baseline between 48 and 96 hours after angiography.

**Discussion:**

The first patient entered the trial on September, 2008. Up to April 7, 2009, 810 patients had been included in 35 centers. The mean age was 69 (Standard deviation: 10), 18% had a baseline serum creatinine >1.5 mg/dL, 57% were diabetics and 13% had a history of heart failure. The ongoing ACT Trial is the largest multicentre RCT that will determine whether acetylcysteine is effective in decreasing the risk of CIN in patients at risk undergoing angiography.

**Trial registration:**

Clinicaltrials.gov NCT00736866

## Background

Contrast-induced nephropathy (CIN) is a potentially serious complication of diagnostic and therapeutic procedures requiring intravenous administration of radiocontrast media. CIN is the third most common cause of new acute renal failure in hospitalized patients and is associated with the need of dialysis, prolonged hospital stay, increased health care costs and death[1,2]

The incidence of a rise in the serum creatinine of more than 50% above baseline or of more than 1 mg/dL is negligible in patients with normal renal function[3] However, patients with previous risk factors such as preexisting renal failure, diabetes, congestive heart failure, advanced age or concurrent administration of nephrotoxic drugs are at a much increased risk. [4] The incidence of CIN has been reported as 9 to 38% in patients with mild to moderate renal insufficiency and diabetes mellitus. [3,5] Patients requiring coronary intervention are another group at increased risk, with an overall incidence of acute renal failure (defined as a 25% increase in serum creatinine above baseline) of 14.4%. [2]

Optimal care to prevent CIN remains uncertain. [6] Patients with normal renal function and no other risk factors are at little risk and few measures are needed except for avoidance of volume depletion. For patients at increased risk the most well established measures are: 1) Avoidance of high osmolal agents[7]; 2) Avoidance of larger radiocontrast doses and repetitive studies (less than 48 hours apart); 3) Volume expansion with isotonic fluids[8] The infusion of isotonic sodium bicarbonate has been shown to be superior to isotonic saline to prevent CIN in most, but not all, randomized controlled trials. [9-12]

The role of acetylcysteine in preventing CIN has been evaluated in several randomized controlled trials and systematic reviews, being the most widely studied of all prophylaxis strategies. [13-16] Previous trials have low statistical power (median study size was 80 patients) and most failed to met quality standards such as allocation concealment, blinding, and intention-to-treat analysis. Similarly, systematic reviews and meta-analysis have found conflicting results. The most recent review, published by *Kelly et al*., included 26 randomized trials and found a protective effect of acetylcysteine (relative risk reduction of 38%), but with significant heterogeneity between the included trials (I^2 ^of 55%), meaning that 55% of the heterogeneity found could not be explained by chance alone. [16] This quite significant heterogeneity among studies' results prevents definitive conclusions to be drawn. An attempt to reveal the causes of heterogeneity using unsupervised clustering analysis revealed that a few smaller trials of average lower quality had similar very favorable results. [15] Removing these studies would leave another cluster of homogeneous trials with adequate quality showing neutral effect of acetylcysteine for preventing CIN. Besides methodological quality, differences in contrast media, outcome definitions, patient selection, dose and route of administration of acetylcysteine, and co-interventions may have contributed to the observed heterogeneity between trials.

Thus, the available evidence on the effects of acetylcysteine does not allow definitive conclusions about the efficacy of acetylcysteine for prevention of CIN. Moreover, it is not clear from previous evidence, what is the most effective dose of acetylcysteine, and what population benefits most from this intervention. A large randomized controlled trial, with high methodologic quality, involving several centers and with sufficient power to evaluate clinically relevant outcomes is needed to ultimately resolve whether acetylcysteine prevents CIN.

The ACT Trial is a randomized controlled trial to determine whether acetylcyteine reduces the risk for CIN in 2300 at-risk patients (ie, with previous renal failure or with other risk factors for contrast-induced nephropathy) undergoing an intravascular angiographic procedure. CIN is defined as an increase of 25% in serum creatinine measured between 48 to 96 hours compared to baseline. The ACT represents the largest trial conducted so far in this field.

## Methods

### Study Design

ACT is a randomized (concealed) controlled trial of acetylcysteine versus placebo in patients at-risk for CIN undergoing an intravascular angiographic procedure. Participants, health care staff, data collectors, outcome assessors, and statisticians will be blinded to whether patients receive acetylcysteine or placebo. All analysis will follow the intention-to-treat principle.

### Eligibility

The study population is comprised of patients at increased risk for CIN undergoing an angiographic procedure (coronary or peripheral arterial diagnostic intravascular angiography or percutaneous intervention). Therefore, risk factors for CIN such as previous renal failure, diabetes mellitus, heart failure, shock states and age greater than 70 years old were selected as the ACT study's inclusion criteria. [6] The Appendix presents the ACT trial's inclusion and exclusion criteria.

### Randomization and Allocation Concealment

Patients are randomized after providing a written informed consent in a 1:1 ratio to receive acetylcysteine or matching placebo. The random allocation list was generated in random permuted blocks of variable size (4, 6, 8 or 10) and was stratified by investigator centre. In order to guarantee concealment of the allocation list, randomization is implemented through a 24-hour web-based automated randomization system, making the sequence allocation totally unpredictable for the professionals responsible for entering the patients in the trial in each centre. The randomization list was generated and implemented by the The ACT study Clinical Data Management System (CDMS), a web based system that has been developed on a Microsoft SQL^® ^platform by a team of programmers of the Research Institute – Hcor, Sao Paulo, Brazil.

### Interventions

#### Study Drugs

**Figure 1:**
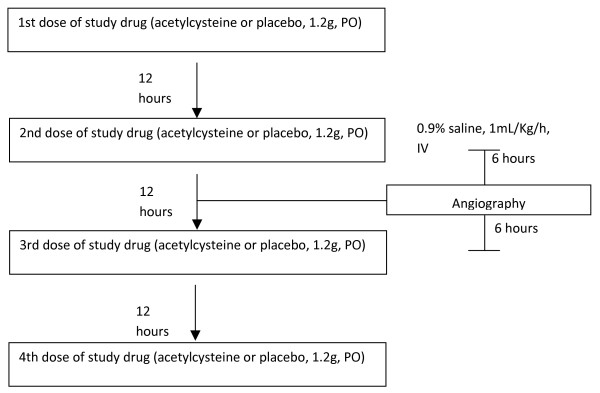
**Flowchart of study drug administration**. Angiography may be done at any time between 6 hours after the first study drug dose to just before the 3^rd ^study drug dose.

The study drugs (acetylcysteine 1200 mg or matching placebo) will be administered orally twice daily for two doses before the procedure and two doses after the procedure (Figure 1). On occasion, when the patient is included in the study on the same day of the angiography, the pragmatic design of the ACT trial allows one dose to be administered at least 6 hours before the procedure and three doses after it.

#### Cointerventions

Hydration with 0.9% saline, 1 mL/kg/hour, starting 6–12 hours before angiography and continued for 6–12 hours after is strongly recommended for all patients. Changes in the total volume or flow may be needed according to attending physician, for example, for patients with signs of congestive heart failure.

All other decisions about management of patients are at the discretion of the responsible doctor, except that non-trial acetylcysteine will not be allowed during the trial period.

### Blinding

All study participants will be blinded to the assigned treatment, including patients, healthcare personnel, investigators and outcome assessors. The study statistician will be the only person able to access unblinded data, albeit she will not have any contact with participants and will not to carry any analysis according to study group before the database is locked.

The study drug, acetylcysteine or placebo, are packed in identical envelops. The envelop label contains information about the study drug and the treatment number. Each envelop contains 600 mg of oral powder acetylcisteine or placebo to be diluted in water. The powder and the solution in water are identical in appearance, taste and smell. In this regard, both are orange flavored.

### Outcomes

All outcomes of interest will be assessed in a blinded fashion. Data will be obtained at baseline, 48–96 hours and 30 days. The primary outcome of the ACT Trial is the occurrence of contrast-induced nephropathy (CIN), defined as a 25% elevation of serum creatinine above baseline between 48 and 96 hours after angiography. The secondary outcomes are: (1) A composite outcome of death, need of dialysis or a doubling in serum creatinine in thirty days; (2) A composite of death or need of dialysis in thirty days; (3) Individual components of the composite outcome.

### Data Collection and Management

#### Baseline Data

The following data will be recorded at the baseline visit:

• Patient's initials, gender, date of birth;

• Inclusion and exclusion criteria;

• Relevant medical history: hypertension; use of non-steroidal anti-inflammatory within last 7 days; use of angiotensin converting enzyme inhibitors, diuretics, metformin; use of aminoglycosides, vancomycin, and other antibiotics;

• Serum creatinine: The most recent serum creatinine measured within the last three months will be recorded. When a recent creatinine measure is not available, a blood sample measurement will be drawn before angiography;

• Physical exam: arterial blood pressure and weight;

• Whether the patient undergone an angiographic procedure in the last 72 hours; if yes, the purpose (diagnosis or treatment), and whether any measure to prevent CIN was provided.

#### Treatment Data

The following data regarding treatment will be obtained:

• Administration of the first and second study drug doses; with details regarding timing and amount per dose;

• Hydration scheme before angiographic procedure: type of solution, total volume and duration of infusion;

• Angiographic procedure: date, hour and type (coronary or peripheral; diagnostic or therapeutic; if therapeutic, angioplasty, bare metal stent, drug-eluted stent or a combination of them;

• Contrast: type, volume administered;

• Whether the procedure was indicated for a patient with acute coronary syndrome.

#### Short-Term Follow-up Data

This is the main follow-up visit. Patients will have a serum creatinine measured between 48 and 96 hours after angiography for evaluation of the study primary outcome. Other data elements which will be recorded are:

• Administration of the third and fourth study drug doses; with details regarding timing and amount per dose;

• Hydration scheme after angiographic procedure: type of solution, total volume and duration of infusion;

• Whether the patient undergone an angiographic procedure within 72 hours after the index angiography; if yes, date, type, and measures to prevent CIN;

• Vital status; in case of deaths, date and cause;

• Need of dialysis.

#### One month follow-up data

Research personnel will contact patients 30 days after the angiographic procedure and record the occurrence of:

• Angiographic procedure: date, type, measures to prevent CIN;

• Vital status; in case of deaths, date and cause;

• Need of dialysis.

#### Adverse events form

This form contains questions for adverse events which may occasionally occur after acetylcysteine (nausea, emesis, urticaria, bronchospasm), open questions for registration of any other adverse event, and a question for severe adverse event with a field for detailing the event.

#### Clinical Data Management System (CDMS) and Quality Control

The ACT study CDMS is a web based system that has been developed on a Microsoft SQL^® ^platform by a team of programmers of the Research Institute – HCor. Its functionalities include: patient registration, 24-hour concealed randomization, data entry, data cleaning and exportation for analysis.

The CDMS also provides reports on the status of the study forms (completed forms, overdue forms), weekly study recruitment by center and graphs of observed and expected cumulative recruitment.

### Sample size

Based on a recent systematic review and meta-analysis of 26 randomized controlled trials, we anticipate an incidence of CIN at 48 to 96 hours of approximately 15%[16] In order to detect a 30% relative risk reduction (RRR), with 90% statistical power, and a two-tailed alpha of 5%, we will need to include at least 2,300 patients.

### Statistical Analysis Plan

All analysis will follow the intention-to-treat principle. The effect of acetylcysteine versus placebo on the primary endpoint, incidence of CIN between 48 to 96 hours, will be evaluated with a chi-square test. The magnitude of association will be presented as a risk ratio, with 95% confidence interval and number needed to treat (NNT). Statistical significance will be inferred using a significance level of 0.05.

Secondary outcomes evaluated 30 days after randomization will be presented as Kaplan-Meier curves according to the assigned treatment, and tested with the log-rank method. Hazard ratios and 95% confidence intervals will be calculated using non-adjusted Cox proportional hazards.

The incidence of the primary outcome will be analyzed in pre-specified subgroups using risk ratios and 95% confidence intervals. A subgroup effect will be inferred when the chi-squared test for homogeneity of effects is statistically significant. The following subgroups will be analyzed: 1) ≤70 year-old versus >70 year-old; 2) Male vs. female; 3) No previous renal failure vs. previous renal failure (serum creatinine >1.5 mg/dL); 4) Not diabetic vs diabetic; 5) Amount of contrast agent <140 mL vs. ≥140 mL. [17]

In the main trial publication, we also plan to include a random effects (DerSimonian-Laird) meta-analysis to combine ACT's results with those of previous randomized controlled trials evaluating acetylcysteine versus placebo for prevention of contrast-induced nephropathy. [18]

### Ethical Aspects

Each study site will submit the study protocol to its institutional Research Ethics Board (REB). The study should start only after being approved by the REB. Written informed consent will be obtained from all participants. This study is in compliance with the Helsinki Declaration.

### Trial Organization and Management

#### Trial Management Team (TMT)

A team based on the Research Institute -Hcor, São Paulo, Brazil, will manage the trial on a day-to-day basis. The TMT is comprised by the chief investigator, a project manager, a clinical research associate, a statistician and four computer programmers. Data management is assumed by both the medical project manager and the clinical research associate.

The responsibilities of the TMT include:

- Selecting and training participant centers;

- Assisting trial centers with regulatory submissions;

- Distributing and supplying study sites with the study drug and forms;

- Monitoring recruitment and follow-up at participant centers;

- Data management: Elaboration, testing and maintenance of the electronic data capture system; data quality control;

- Data analysis;

- Servicing the Trial Steering Committee.

#### Trial Steering Committee (TSC)

The TSC is responsible for providing overall supervision of the trial, assist with development of the study protocol and preparing the final manuscript. All other trial's committees report to the TSC. The TSC members are epidemiologists, with in-dept training in designing and conducting randomized controlled trials, or cardiologists experienced in the conduction of multicentre clinical trials.

#### Trial Centers

Fifty four invasive catheterization centers in Brazil are participating in the study. Details of the participating centers are given in the Appendix.

#### Publication policy

The ACT study success depends on all its collaborators. Therefore, the primary results of the trial will be published under the name of ACT Trial Investigators. The contributions of all collaborators, their names and respective institutions, not only the members from TSC or TMT, will be acknowledged in the manuscript. To safeguard the scientific integrity of the study, data from this study will be submitted to publication only after the final approval from the TSC.

#### Data Monitoring Committee

The ACT Study does not have a Data Monitoring Committee, nor will we conduct interim analysis to evaluate drug efficacy or safety. The arguments supporting this decision are: 1) Oral acetylcysteine has a very good safety profile, being tested in several small clinical trials and used in practice for a long period of time. Adverse reactions (specially unexpected severe adverse events) and drug interactions are uncommon and rarely severe. [19] Therefore, early stopping for safety reasons would be highly unlikely. 2) Trials stopped early for benefit usually reach large and non-plausible treatment effects (relative risk reductions greater than 50%) due to low numbers of events and thus low statistical power to reach robust results. In consequence, several experts in the field of clinical trials have criticized and discouraged such an approach. [20] 3) The study has limited power to evaluate clinical events such as need of dialysis or death. We expect an incidence of need of dialysis or death in 30 days between 2% and 3%. A study enrolling 7,650 patients would be needed to have 80% power to show a reduction in this combined outcome from 3% to 2%. Therefore, it is very unlikely that evidence of benefit beyond doubt on clinical outcomes would be found in an interim analysis.

## Discussion

Contrast-induced nephropathy is a common complication after coronary or peripheral angiography. Patients at increased risk include those with previous renal failure, diabetes mellitus, heart failure, shock states and elderly. [4] Acetylcystine represents a simple, non-toxic, low-cost, and wide available intervention. The majority of previous trials that tested this intervention had small sample sizes, inadequate methodology, have reached conflicting results, and have not assessed patient-important outcomes. At present there is limited evidence that acetylcysteine together with hydration may be useful as standard prophylactic procedure in patients at high risk for CIN. Therefore, a well designed trial with adequate statistical power is needed to resolve the question of acetylcysteine for the prevention of CIN.

ACT investigators started recruitment on September, 15, 2008. To date (April 7, 2009), 35 centers started recruitment and 810 patients have been randomized. Thus, ACT is already the largest randomized controlled trial evaluating a strategy to prevent CIN[16] Patients' baseline characteristics are depicted in table 1. Mean age was 69 (standard deviation 10), with 57% being older than 70 years. Prevalence of renal failure (as defined by a baseline serum creatinine > 1.5 mg/dL) was 18%, of diabetes was 57% and of heart failure was 13%.

**Table 1:** Baseline demographic and clinical characteristics

**Characteristic**	**Total** **(N = 810)**
Female sex – no. (%)	305 (38)
Age – years	
Mean (SD)	69 (10)
Patients fulfilling inclusion criteria – no. (%)	
Recent creatinine >1.5 mg/dL	148 (18)
Diabetes mellitus	458 (57)
Heart failure	105 (13)
Shock	2 (0.2)
Aged >70 years	458 (57)
History of hypertension – no. (%)	659 (81.4)
Serum creatinine – mg/dL	
Median (IQR)	1.1 (0.5)
Estimated creatinine clearance* – mL/kg/min	
Median (IQR)	60 (38)

SD, standard deviation; IQR, interquartile range.

* Creatinine clearance estimated by the Cockcroft-Gault formula.

The ACT study was planned to be the largest, double-blind, randomized controlled trial, comparing acetylcysteine to placebo in high risk patients undergoing peripheral or cardiac invasive angiography conducted to date. The results of ACT will convey a reliable and precise estimate of acetylcysteine's effectiveness on preventing CIN in such population. Regardless of the results, the ACT trial will have important implications for clinical practice. If the ACT trial demonstrates no effect on contrast induced nephropathy and on other patient-important outcomes, then it will allow physicians to avoid unnecessary patient risk and decrease costs. On the other hand, if the ACT Trial demonstrates a beneficial effect of acetylcysteine, then it will have clinical impact on preventing renal adverse events in high risk patients undergoing angiographic procedures.

## Competing interests

The authors declare that they have no competing interests.

## Authors' contributions

The ACT Trial Investigators contributions are listed in the Acknowledgements. All the investigators at study sites participated in the planning phase of the study, besides their contributions in enrolling and following patients. The Writing Committee elaborated the article and assumes full responsibility for its overall content and integrity.

## Appendix

### Eligibility criteria of the ACT Trial

#### Inclusion criteria

Patients undergoing an angiographic procedure must meet at least one of the following criteria to be included:

• Age > 70 years;

• Renal failure (defined as a serum creatinine higher than 1.5 mg/dL within the last 3 months);

• Diabetes mellitus;

• Congestive heart failure or left ventricular ejection fraction <0.45;

• Shock;

• Intra-aortic balloon counterpulsation.

### Exclusion criteria

Patients are excluded if they met any of the following criteria:

• Pregnant or breastfeeding women, or aged below 45 years and with no efficacious contraceptive method;

• Patients on dialysis;

• Previous enrollment in ACT study;

• Patient did not provide informed consent;

• Patients with ST segment elevation myocardial infarction unable to receive the study hydration protocol.
